# Immunohistochemical and Ultrastructural Evaluation of Human Fetal Testicular Development Across Different Gestational Ages

**DOI:** 10.7759/cureus.113194

**Published:** 2026-07-22

**Authors:** Arpan Haldar, Manisha R Gaikwad, Apurba Patra

**Affiliations:** 1 Anatomy, All India Institute of Medical Sciences, Deoghar, IND; 2 Anatomy, All India Institute of Medical Sciences, Bhubaneswar, IND; 3 Anatomy, All India Institute of Medical Sciences, Bathinda, IND

**Keywords:** leydig cells, macrophages, sertoli cells, spermatogonia, steroidogenic cells

## Abstract

Introduction

Human fetal testicular development is a tightly regulated process involving coordinated cellular differentiation and structural maturation. However, comprehensive immunohistochemical and ultrastructural characterization across different gestational ages remains limited, restricting a detailed understanding of normal testicular development.

Materials and methods

Testicular tissue samples obtained from aborted human fetuses of varying gestational ages were studied. The tissues were subjected to Ki-67 antibodies for immunohistochemistry, examined through scanning electron microscopy (SEM) and transmission electron microscopy (TEM) to assess cellular organization and ultrastructural features.

Results

The fetal testis consisted of solid seminiferous (testis) cords lacking a central lumen, with no distinct basal and adluminal compartments due to the absence of Sertoli cell tight junctions and the blood-testis barrier. The cords were lined by immature Sertoli cells enclosing centrally located gonocytes. The interstitial tissue contained abundant fetal Leydig cells arranged singly or in small clusters. Ultrastructurally, fetal Leydig cells exhibited abundant smooth endoplasmic reticulum, numerous mitochondria with tubular cristae, prominent Golgi complexes, and lipid droplets, consistent with their active steroidogenic function during fetal development.

Conclusion

Human fetal testicular development is characterized by progressive structural organization of seminiferous cords and differentiation of immature Sertoli and fetal Leydig cells. The immunohistochemical and ultrastructural findings demonstrate the establishment of the cellular architecture required for future spermatogenesis while confirming the active steroidogenic role of fetal Leydig cells during gestation. These observations provide a comprehensive baseline for understanding normal fetal testicular development and may serve as a reference for investigating disorders of testicular differentiation and development.

## Introduction

The human seminiferous epithelium demonstrates a highly organized process of spermatogenesis in which germ cells at different developmental stages form distinct cellular associations. Tight junctions between Sertoli cells divide the seminiferous epithelium into basal and adluminal compartments, thereby regulating germ cell maturation [1]. Spermatogonia located in the basal compartment undergo mitotic and meiotic divisions to form mature spermatozoa through spermiogenesis [2]. Advanced studies using computer modeling have demonstrated that germ cell distribution follows a precise helical arrangement rather than an irregular pattern. The intertubular tissue contains steroid-producing Leydig cells, characterized by abundant smooth endoplasmic reticulum (sER), mitochondria with tubular cristae, and Reinke crystalloids [3-5]. Previous studies have reported pyknotic changes, depletion of Leydig cells, Sertoli cell vacuolization, mitochondrial alterations, and increased oxidative stress following exposure to anabolic steroids and toxic agents, resulting in germ cell apoptosis and sloughing [6]. The ultrastructural features of the human testis are potentially crucial in understanding seminoma testis, Leydig cell tumors, and testicular tissue xenografting [7]. Hence, the present study was undertaken to investigate the immunohistochemical and ultrastructural organization of the human testis to establish a morphological reference that may facilitate future studies involving testicular development and pathologies.

## Materials and methods

This was an observational descriptive study conducted on human fetal testicular tissue samples collected from therapeutically aborted fetuses between 2017 and 2018. The study was carried out in the Department of Anatomy in collaboration with the Department of Obstetrics and Gynecology, All India Institute of Medical Sciences, Bhubaneswar. A total of 10 fetal testicular tissue samples representing different gestational periods (weeks) of the first, second, and third trimesters were included in the study.

The gestational age of each fetus was determined based on maternal history, ultrasonographic findings, and fetal biometric parameters. The collected samples were categorized according to trimester to evaluate developmental changes in testicular morphology, proliferative activity, and ultrastructural characteristics.

Inclusion and exclusion criteria

Fetal testicular tissue samples obtained from therapeutically terminated pregnancies were included in the study after obtaining written informed consent from the legal guardians. Fetuses were included if the fetus had a confirmed male phenotype, testicular tissue was grossly identifiable and adequate for histological and ultrastructural evaluation, and the fetus had no evidence of congenital anomalies affecting the reproductive system. Samples were excluded if external congenital abnormalities were present; there was evidence of cryptorchidism, testicular agenesis, or gross developmental abnormalities of the testes; the tissue sample was inadequate or poorly preserved for immunohistochemistry or electron microscopy analysis; and there was evidence of maceration or autolysis of fetal tissue.

Ethical considerations

The study was approved by the Institutional Ethics Committee and Institutional Review Board of All India Institute of Medical Sciences, Bhubaneswar (Approval No. T/IM-NF/Anatomy/18/84). Written informed consent was obtained from the parents/legal guardians prior to sample collection. All procedures were performed according to institutional ethical guidelines and the principles of the Declaration of Helsinki.

Sample size

The sample size was determined based on the availability of fetal testicular specimens during the study period and the feasibility of detailed histological, immunohistochemical, and ultrastructural analysis. As this was an exploratory morphological study involving human fetal tissue, a purposive sampling method was adopted. A total of 10 samples distributed across the first, second, and third trimesters were analyzed to identify developmental variations in testicular architecture and cellular proliferation.

Study parameters

The following parameters were evaluated: (1) histomorphological assessment (general architecture of fetal testicular tissue, arrangement and morphology of seminiferous cords/tubules, morphological characteristics of Sertoli cells and germ cells, and presence and appearance of interstitial Leydig cells); (2) immunohistochemical assessment (expression of Ki-67 as a marker of cellular proliferation, percentage of Ki-67-positive cells among testicular cell populations, and comparison of proliferative activity between different gestational periods); and (3) ultrastructural assessment (surface morphology of testicular components using scanning electron microscopy (SEM) and cellular ultrastructure and organelle characteristics using transmission electron microscopy (TEM).

Immunohistochemistry

Testicular tissue samples were fixed in 10% neutral buffered formalin, routinely processed, and embedded in paraffin wax. Sections of approximately 3 µm thickness were prepared and subjected to heat-induced antigen retrieval using Danish Antibody Company (DAKO) Target Retrieval Solution.

Sections were incubated with Ki-67 primary antibody, followed by detection using a labeled avidin-biotin immunoperoxidase technique on a DAKO Auto Stainer. Diaminobenzidine (DAB) was used as the chromogen, and hematoxylin was used for counterstaining. Ki-67 immunoreactivity was evaluated semi-quantitatively according to the percentage of positive cells and graded as 0: no positive cells; 1+: <10% positive cells; 2+: 10%-25% positive cells; 3+: 26%-50% positive cells; 4+: >50% positive cells.

Scanning electron microscopy

Small pieces of fetal testicular tissue were fixed in phosphate-buffered fixative followed by postfixation with osmium tetroxide. Samples were dehydrated through ascending grades of acetone and subjected to critical point drying using liquid carbon dioxide. The dried specimens were mounted on aluminum stubs, sputter-coated with gold, and examined under a scanning electron microscope for surface ultrastructural evaluation.

Transmission electron microscopy

For ultrastructural evaluation, tissue fragments measuring approximately 2 × 2 mm were fixed in Karnovsky’s fixative followed by postfixation in osmium tetroxide. The samples were dehydrated through graded alcohol concentrations and embedded in epoxy resin. Ultrathin sections (50-70 nm) were prepared using an ultramicrotome, stained with uranyl acetate and lead citrate, and examined under a transmission electron microscope.

Statistical analysis

As the study was exploratory in nature with a limited sample size, formal inferential statistical analysis was not performed. Observations were described qualitatively and semi-quantitatively.

## Results

A total of 10 fetal testicular tissue samples representing the first, second, and third trimesters of gestation were examined. Progressive developmental changes in the testicular architecture, Leydig cell morphology, and immunoreactivity pattern were observed with increasing gestational age.

During the first trimester, the fetal testis demonstrated prominent interstitial cellularity with abundant Leydig cells arranged as clusters or broad cellular sheets between the developing seminiferous cords. Leydig cells showed intense cytoplasmic immunoreactivity, indicating high expression of the studied marker. The immunopositive Leydig cells were distributed throughout the interstitial compartment.

The developing seminiferous cords showed cytoplasmic immunolabeling involving germ cells and Sertoli cells, producing distinct dark cord-like structures. Compared with Leydig cells, tubular cells exhibited relatively weaker staining intensity. Occasional nuclear positivity was observed in both Leydig cells and tubular components.

The epididymal tissue associated with fetal testis also demonstrated immunoreactivity, particularly involving the apical cytoplasm of epididymal epithelial cells. Numerous immunopositive cells were identified within the fetal testicular tissue during this developmental period. Ki-67 immunostaining demonstrated nuclear positivity predominantly in the epithelial cells of the developing epididymal ducts, with occasional positive stromal cells, consistent with active fetal tissue proliferation.

During the second trimester, Leydig cells demonstrated morphological maturation and appeared predominantly rounded or polygonal in shape. Most Leydig cells exhibited strong cytoplasmic immunoreactivity, although occasional weakly stained or negative cells were observed.

The solid seminiferous (testis) cords showed progressive development with comparatively reduced immunostaining intensity compared with the interstitial Leydig cells. The proportion of immunoreactive cells appeared decreased relative to the first trimester, corresponding with increased seminiferous tubular development and reduction of relative interstitial tissue area.

**Figure 1:**
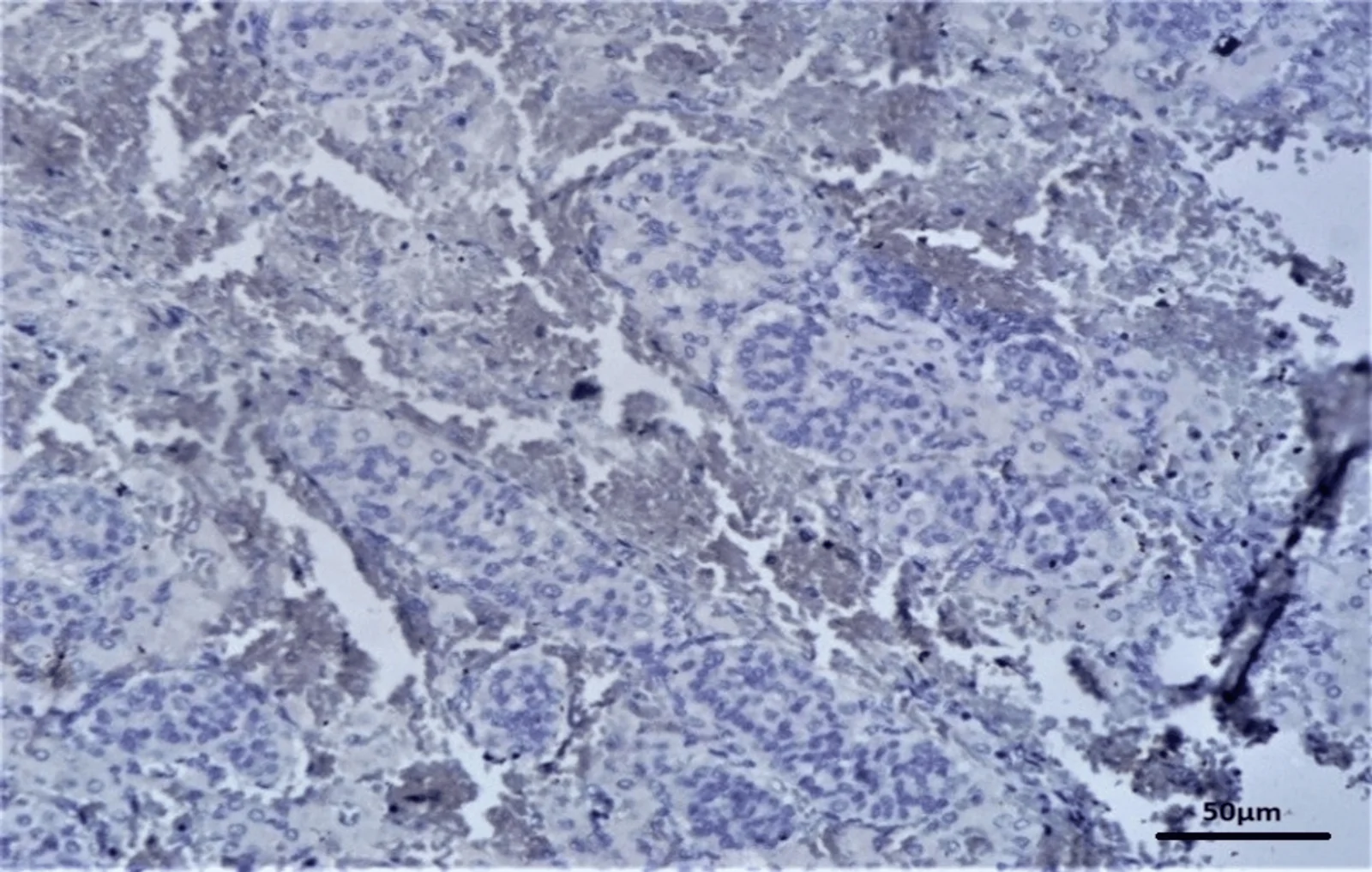
40x magnification showing deep surface of testis Ki-67-immunoreactive cells are less numerous, and Leydig cells are slightly flattened due to reduced space within the interstitial tissue

In the third trimester, Leydig cells appeared more compact and slightly flattened, corresponding to a reduction in the available interstitial space. The intensity of Leydig cell immunoreactivity was variable, with mature Leydig cells showing stronger staining while some immature/regressed Leydig cells demonstrated weak or absent immunolabeling (Figure 1).

**Figure 2:**
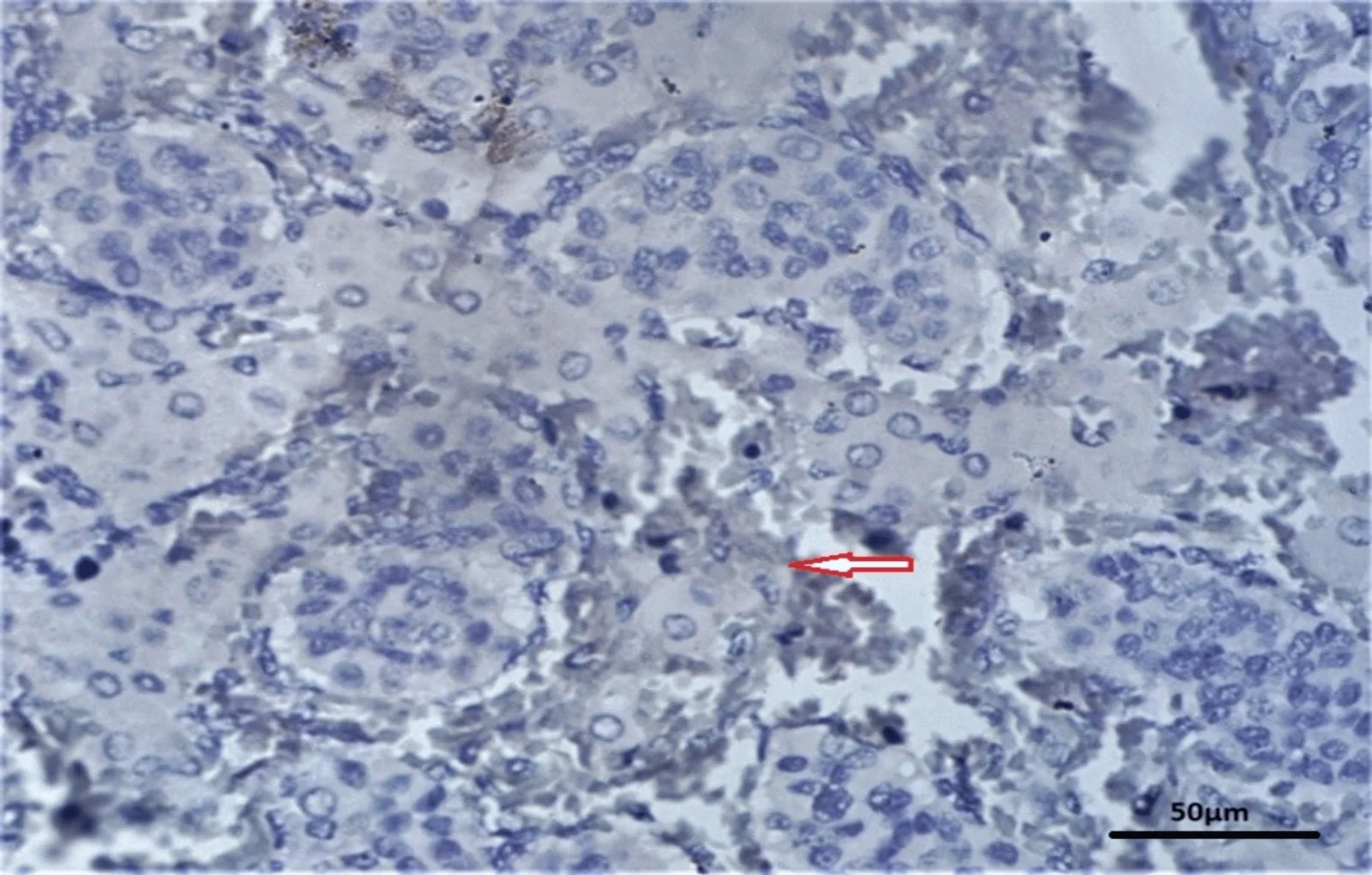
40x showing high magnification of deep surface The epididymis tubular cells show strong Ki-67 immunoreactivity

The epididymal epithelium continued to demonstrate strong cytoplasmic immunoreactivity (Figure 2). A distinct reduction in the number of strongly immunoreactive Leydig cells was observed compared with earlier gestational periods.

Overall, Ki-67 immunoreactivity progressively decreased with advancing gestational age, reflecting reduced cellular proliferation as the fetal testis underwent maturation.

Ultrastructural findings

**Figure 3:**
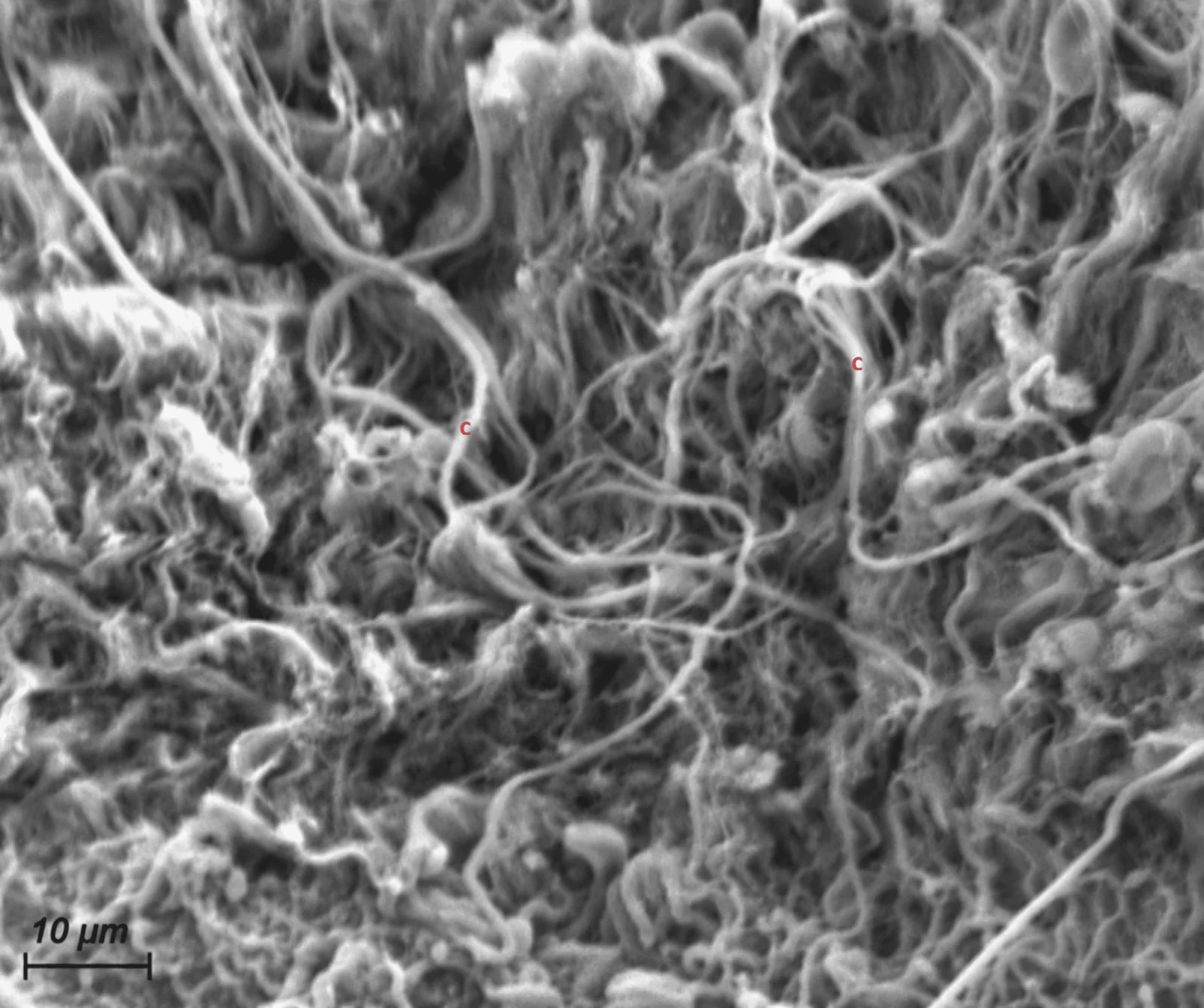
Scanning electron microscope Showing surface of seminiferous cords (C)

During the first trimester of gestation, the testicular interstitium showed fibroblast-like interstitial cells along with fetal Leydig cells. Leydig cells exhibited characteristic steroidogenic features, including abundant sER, mitochondria with tubular cristae, lipid droplets, and electron-dense bodies. Degenerating Leydig cells showed dark cytoplasm, reduced sER, residual bodies, and lipid accumulation. Gonocytes displayed a prominent nucleolus, dispersed chromatin, Golgi apparatus, mitochondria, and polyribosomes. Chromatid bodies were also observed in intermediate cells (Figure 3).

**Figure 4:**
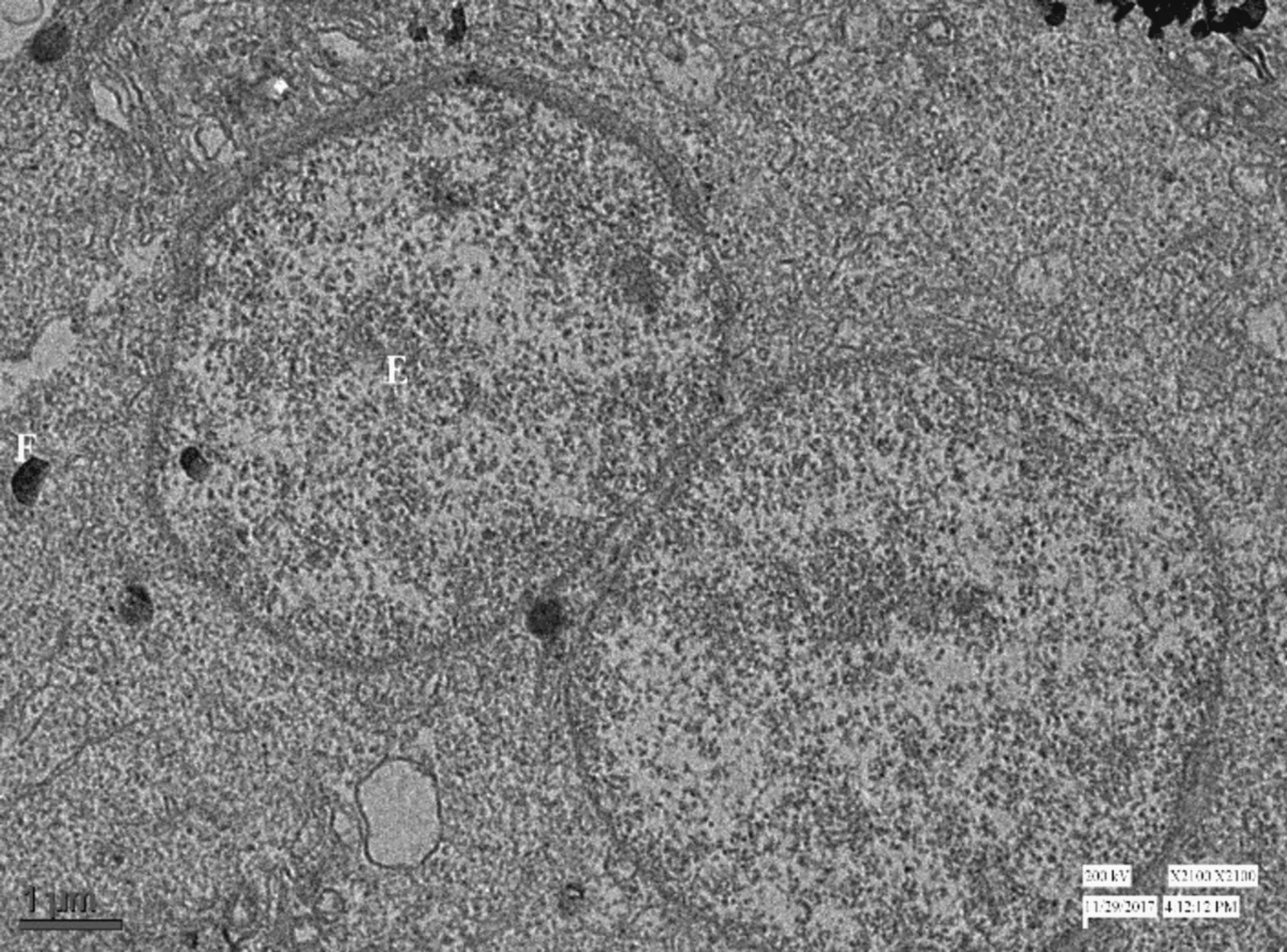
Transmission electron microscopy The figure shows the nucleus of Sertoli cells with an intact nuclear membrane (E) and dense body in the cytoplasm of fetal germ cells or gonocytes (F)

In the second trimester of gestation, the interstitium contained numerous fetal Leydig cells with dark cytoplasm and lipid vacuoles, surrounded by fusiform peritubular cells. Immature Leydig cells and mesenchymal cells were observed in the intermediate region. Blood vessels and basement membranes showed strong immunoreactivity, while primitive spermatogonia and immature Sertoli cells showed positivity. Undifferentiated mesenchymal cells demonstrated triangular nuclei with scant organelles and elongated cytoplasmic processes (Figure 4).

**Figure 5:**
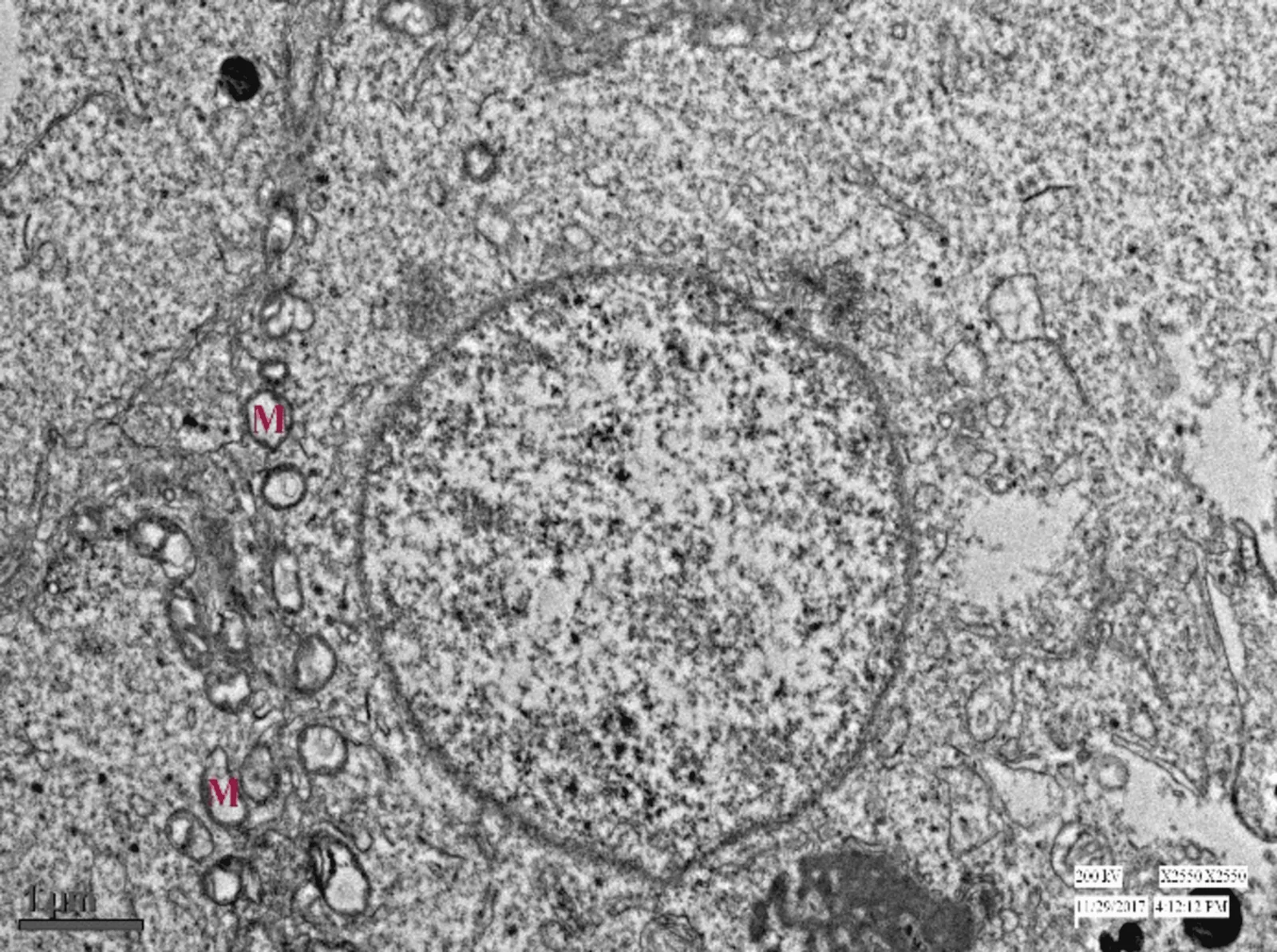
Transmission electron microscopy Normal Leydig cells with no cytoplasmic vacuolation (M) are visible

By the third trimester of gestation, the lamina propria consisted of one to two layers of flattened peritubular cells. Elongated myoid-like cells showed strong cytoplasmic immunoreactivity, with primitive spermatogonia and immature Sertoli cells evident within the developing testicular cords (Figure 5).

TEM and SEM provided a detailed visualization of the organization and interactions of Sertoli cells and Leydig cells during fetal testicular development.

## Discussion

The present study evaluated the morphological, immunohistochemical, and ultrastructural characteristics of human fetal testicular tissue during different gestational periods. Progressive changes in testicular architecture, Leydig cell morphology, and cellular proliferative activity were observed with advancing gestational age. Human fetal testicular development involves coordinated differentiation of Sertoli cells, germ cells, and Leydig cells, with dynamic changes in hormonal activity and tissue organization during gestation [8].

Fetal Leydig cells play a critical role in male sexual differentiation through testosterone production, which regulates the development of the internal and external male reproductive system. In the present study, Leydig cells were prominently distributed within the interstitial compartment during early gestation and demonstrated strong cytoplasmic immunoreactivity, suggesting increased functional activity during this developmental phase. Similar observations have been reported in human fetal testes, where Leydig cell differentiation and steroidogenic activity are prominent during early fetal life, followed by progressive maturation of the interstitial compartment [9,10].

The reduction in Leydig cell immunoreactivity observed during later gestational periods in the present study may represent a physiological developmental transition rather than degenerative change. With advancing gestational age, seminiferous cords become progressively organized and occupy a larger proportion of the testicular parenchyma, resulting in relative reduction of interstitial cellularity. Previous studies have demonstrated progressive Sertoli cell differentiation and seminiferous cord maturation during fetal development [11].

Ki-67 immunohistochemistry was performed to evaluate proliferative activity in developing fetal testicular tissue. Strong immunoreactivity was observed in fetal epididymal epithelial cells and developing seminiferous structures, indicating active cellular proliferation during organ maturation. Ki-67 is a widely accepted marker of cellular proliferation and reflects active progression through the cell cycle [12]. The observed Ki-67 expression supports ongoing cellular expansion and differentiation during fetal testicular development.

Ultrastructural evaluation by SEM and TEM demonstrated preserved testicular organization without significant degenerative changes. Developing seminiferous cords and interstitial components showed maintained cellular integrity with progressive maturation of cellular components. Previous ultrastructural studies have described similar developmental changes in fetal human testes, including maturation of Leydig cells, Sertoli cells, and germ cells with characteristic alterations in cellular morphology and organelle distribution [12].

Although the present study was not designed to investigate toxic injury, previous studies have described cytoplasmic vacuolization of Sertoli cells as a morphological alteration associated with cellular stress and altered Sertoli cell function under experimental conditions [13,14]. Our observations provide a broader context for interpreting similar histological changes without implying a comparable pathogenic mechanism in the fetal testes examined in the present study.

The close association of Leydig cells with the vascular compartment may increase their susceptibility to circulating toxicants and endocrine-disrupting agents. Multivacuolated Leydig cells have been considered a morphological indicator of cellular stress or involution [9]. Alterations in peritubular myoid cells and extracellular matrix components may influence seminiferous tubular function. Increased collagen deposition and basement membrane thickening have been associated with disruption of seminiferous architecture and impaired spermatogenesis [15].

However, in contrast to these pathological observations, the fetal testicular tissue examined in the present study showed preserved architecture and absence of significant degenerative ultrastructural alterations. The findings suggest normal developmental progression rather than cellular injury. Understanding normal fetal testicular morphology is essential for distinguishing physiological maturation from developmental disorders such as cryptorchidism, testicular dysgenesis syndrome, and testicular tumors [16,17].

The present study provides detailed morphological and ultrastructural information regarding fetal testicular development in an Eastern Indian population. Such baseline information may contribute to future investigations related to reproductive biology and fertility preservation. Human fetal testicular tissue has been explored as an experimental model because of its developmental plasticity and vascular characteristics that may support maturation of germ cells after transplantation [18,19].

Limitations of the study

The present study has certain limitations. The sample size was limited because of restricted availability of ethically obtained human fetal testicular tissue. Therefore, larger studies involving a greater number of samples across different gestational periods are required to validate these findings. The study was primarily based on morphological, immunohistochemical, and ultrastructural evaluation; therefore, functional parameters such as fetal testosterone concentration, molecular markers of differentiation, and gene expression analysis could not be assessed. Additionally, quantitative stereological analysis of individual testicular cell populations was not performed. Despite these limitations, the present study provides important baseline information regarding normal human fetal testicular development.

## Conclusions

This preliminary observational study provides ultrastructural descriptions of the developing human fetal testis, including the morphology of Leydig cells, Sertoli cells, and fetal germ cells, thereby contributing to the limited available data on normal fetal testicular development. These observations may serve as a morphological reference for future studies investigating normal and abnormal testicular development. Further research involving larger sample sizes and molecular techniques will be required to better understand the mechanisms underlying testicular development and associated pathological conditions.
